# The Prevalence and Clinical Significance of Congenital Anomalies of the Kidney and Urinary Tract in Preterm Infants

**DOI:** 10.1001/jamanetworkopen.2022.31626

**Published:** 2022-09-14

**Authors:** Thomas Hays, Michaela V. Thompson, David A. Bateman, Rakesh Sahni, Veeral N. Tolia, Reese H. Clark, Ali G. Gharavi

**Affiliations:** 1 Division of Neonatology, Department of Pediatrics, Columbia University Irving Medical Center, New York, New York; 2 Department of Pediatrics, Columbia University Irving Medical Center, New York, New York; 3 The MEDNAX Center for Research, Education, Quality and Safety, Sunrise, Florida; 4 Division of Neonatology, Department of Pediatrics, Baylor University Medical Center, Dallas, Texas; 5 Pediatrix Medical Group, Dallas, Texas; 6 Division of Nephrology, Department of Medicine, Columbia University Medical Center, New York, New York

## Abstract

**Importance:**

The prevalence and importance of congenital anomalies of the kidney and urinary tract (CAKUT) in preterm infants is unknown.

**Objective:**

To determine the prevalence of CAKUT in preterm infants and association with in-hospital morbidity and mortality.

**Design, Setting, and Participants:**

This cohort study included infants cared for in neonatal intensive care units managed by a large US network of hospitals and doctors. Eligible participants were infants born at 23 to 33 weeks’ gestation between 2000 and 2020. Infants transferred from or to other health care facilities prior to discharge or death were excluded in analysis of outcomes. Data were analyzed from December 2021 until May 2022.

**Exposures:**

The presence of anomalies of the kidneys, ureters, bladder, or urethra was assessed. Covariates were discharge year, exposure to antenatal steroids, sex, maternal race, gestational age, birthweight, mechanical ventilation in first 72 hours of life, genetic disorders, and extrarenal anomalies.

**Main Outcomes and Measures:**

Death or in-hospital severe illness (acute kidney injury, kidney failure, intracranial hemorrhage, necrotizing enterocolitis, bronchopulmonary dysplasia, bacterial sepsis, or administration of inotrope or vasopressor).

**Results:**

In this cohort of 409 704 infants, 191 105 (46.6%) were girls, mean (SD) gestational age was 30.1 (2.84) weeks, and mean (SD) birth weight was 1.49 (0.53) kg. A total of 8093 infants (2.0%) had CAKUT, with urinary tract dilation comprising the majority of cases (5669 [70.0%]). The presence of CAKUT correlated with earlier gestational age and was associated with genetic disorders and extrarenal anomalies. Analysis of 323 957 infants after exclusions demonstrated an adjusted odds ratio of 3.96 (95% CI, 3.70–4.24) of death or severe illness. This risk was found across all forms of CAKUT including isolated urinary tract dilation.

**Conclusions and Relevance:**

The findings of this cohort study suggest that clinicians caring for preterm infants should have higher suspicion for CAKUT and consider screening, particularly those with extrarenal anomalies or genetic disorders, as preterm infants with CAKUT appear to be at significantly higher risk of death or severe illness. Detection of CAKUT can inform risk stratification and clinical decision making, and should also prompt clinicians to consider a genetic evaluation.

## Key Points

**Question:**

What is the prevalence and importance of congenital anomalies of the kidney and urinary tract (CAKUT) in preterm infants?

**Findings:**

In this cohort study of 409 704 infants born at 23 to 33 weeks’ gestation from 2000 to 2020, 2.0% had CAKUT. CAKUT was associated with prematurity, genetic disorders, and extrarenal anomalies, and was significantly associated with risk of mortality or in-hospital morbidity, even for less severe forms such as isolated hydronephrosis.

**Meaning:**

These results suggest that preterm infants with any form of CAKUT, including isolated urinary tract dilation, are at greater risk of death and severe illness, and may benefit from genetic evaluation.

## Teaser

This cohort study of a large US database of infants cared for in neonatal intensive care units examines the prevalence and clinical outcomes for congenital anomalies of the kidney and urinary tract.

## Introduction

Congenital anomalies of the kidneys and urinary tract (CAKUT) is a diagnostic term encompassing heterogenous anomalies, including changes in the size and location of kidneys, dysplastic changes within the kidney parenchyma, and anomalies of the collecting system, ureters, bladder, and urethra.^1^

The etiology of CAKUT is incompletely understood. Exposure to teratogens, folate, and retinoic acid account for a fraction of cases.^2,3^ There is a strong genetic etiology of CAKUT, with 10% to 25% of cases attributable to genetic disorders.^4,5,6,7^ The relationship between genetic disorders and phenotypic manifestations of CAKUT is highly complex. Individuals with the same genomic abnormalities can manifest with variable forms of CAKUT, and similar phenotypic anomalies can arise from distinct genetic disorders.^4,8^ Genetic disorders associated with CAKUT are frequently associated with extrarenal diseases, such as developmental delays, congenital heart disease (CHD), immunodeficiencies, and endocrine disruption.^1,4^ Population-based studies have estimated the prevalence of CAKUT to be between 4 to over 100 per 10 000 individuals.^9,10,11,12,13,14,15,16,17^

Infants born prematurely also experience a high burden of morbidity and mortality early in life.^18^ While risk factors have been identified (eg, degree of prematurity, birth weight, sex, antenatal steroids), outcomes remain highly variable. Recently, genetic disorders have been found to independently contribute to perinatal disease.^19^ To our knowledge, the prevalence of CAKUT in preterm infants has not been described. Given this background, we analyzed a multicenter, retrospective cohort of preterm infants to determine the prevalence of CAKUT and evaluated its association with morbidity and mortality.

## Methods

This study was approved by the institutional review board of Columbia University with a waiver for informed consent, and this report followed the guidelines for cohort studies as outlined by the Strengthening the Reporting of Observational Studies in Epidemiology (STROBE) reporting guideline. This was a retrospective, multicenter cohort study of infants born prior to 34 weeks’ gestation using the Pediatrix Clinical Data Warehouse (CDW).^20^ Deidentified records from 409 704 infants discharged from 2000 to 2020 were collected. Pediatrix neonatal intensive care units (NICUs) care for approximately 20% of infants born prior to 34 weeks’ gestation in the US. Data from NICU admission to discharge were collected.

We defined infants as having CAKUT if any structural anomaly of the kidney or urinary system was present at birth and recorded as a diagnosis in an individual’s electronic medical record. CAKUT presentations were broadly categorized as abdominal wall defects (AWD) including prune belly syndrome and bladder exstrophy, renal ectopy or fusion (EF), unilateral or bilateral renal agenesis (RA), renal hypoplasia or dysplasia (RHD), urinary tract dilation and anomalies of the urethra or ureters (UTD), forms of CAKUT that were not otherwise specified (not specified), and multiple different forms of CAKUT (multiple). We first determined the prevalence of CAKUT. Then, we performed multiple logistic regression to test for factors associated with the presence of CAKUT. These factors were: phenotypic sex, gestational age, birth weight (as a *z* score normalized within this cohort by gestational age), race (defined by maternal self-declaration as identifying as Black, White, Asian, Hispanic, or other), the presence of genetic disorders (aneuploidy, copy number variant [CNV], or single-gene disorders), and the presence of extrarenal anomalies. Race was included given varying prevalences of CAKUT that have been described in individuals from different backgrounds.^9,10,11,12,13,14,15,16,17^ The prevalence of CAKUT was also assessed by simple linear regression against gestational age. The categorized prevalence of CAKUT in this cohort was compared with available population studies, and the crude odds ratio (OR) for the prevalence of CAKUT was determined.

We next evaluated the association of CAKUT with death or severe illness prior to NICU discharge by multiple logistic regression. To limit ascertainment bias, infants transferred after birth (often because of complex illness) and infants transferred prior to discharge (in which their clinical outcome is unknown) were excluded.^21^ We included the following as covariates in the analysis: discharge year epoch (2000 to 2004, 2005 to 2009, 2010 to 2014, 2015 to 2020), exposure to antenatal steroids, phenotypic sex at birth (female sex was treated as reference given the associated risk of illness in male infants),^22,23^ race (White race was treated as reference given the proportional size), gestational age, birthweight (as a *z* score normalized within this cohort by gestational age), use of mechanical ventilation in the first 72 hours of life, presence of a known genetic disorder, and the presence of extrarenal anomalies.

For this analysis, we defined a combined clinical outcome of death or severe illness. Acute kidney injury (AKI) was defined by a diagnosis of oliguria or anuria (*International Classification of Diseases, Ninth Revision *code 788.5 or *International Statistical Classification of Diseases and Related Health Problems, Tenth Revision (ICD-10)* code R34); end stage kidney disease defined as administration of dialysis; severe intracranial hemorrhage (ICH) defined as stage III or IV intraventricular hemorrhage or periventricular leukomalacia^24^; medically or surgically treated necrotizing enterocolitis (NEC)^25^; severe bronchopulmonary dysplasia (BPD) defined as invasive mechanical ventilation at 36 weeks’ postmenstrual age; severe retinopathy of prematurity (ROP) defined as need for any medical or surgical intervention; culture-positive sepsis defined by any positive blood or urine culture; or shock defined as administration of any vasopressor or inotropic agent.

### Statistical Analysis

To assess diseases infants with CAKUT were at risk for, crude ORs were calculated for death and each severe illness compared between infants with and without CAKUT. Next, to assess the risk of death or severe illness in infants with different forms of CAKUT, crude ORs of the combined outcome were calculated for each category of CAKUT. Then, to assess the risk of disease in infants with CAKUT born at different gestational ages, the rates of death or severe illness were compared between infants with and without CAKUT born at each week of gestation from 23 to 33 weeks. To assess for differences in clinical outcomes by facility, the analyses were repeated nesting individuals within NICU facilities and treating facility as a random effect. For comparison, crude ORs of death or severe illness were also determined for infants with CHD, central nervous system (CNS) anomalies, and anomalies of multiple organ systems. For statistical tests, 2-sided *P* values < .05 were considered statistically significant. Bonferroni adjustments were made to *P* values for tests with multiple comparisons. Analyses were made using RStudio version 2022.02.0 (R Project for Statistical Computing) (eMethods in the Supplement).

## Results

Of 409 704 infants in this cohort, 191 105 (46.6%) were girls; mean (SD) gestational age was 30.1 (2.84) weeks, and mean (SD) birth weight was 1.49 (0.53) kg (Table 1). A diagnosis of CAKUT was found in 8093 (2.0%) of cases. Of the 8093 individuals with CAKUT, the following number of cases were found by category, with the percentage noted in parentheses: AWD, 56 (0.7%); EF, 155 (1.9%); RA, 266 (3.3%); RHD, 509 (6.3%); UTD, 5669 (70%); not specified, 1199 (15%); multiple, 239 (3.0%). The prevalence of CAKUT in this cohort was significantly higher than in recent population studies of term infants, which found prevalence rates from 0.04% to 1.1% (eFigure 1 in the Supplement).^9,10,11,12,13,14,15,16,17^

**Table 1.** Descriptive Characteristics of the Cohort

Characteristics	Patients, No. (%)		*P* value
	CAKUT absent (N = 401 611)	CAKUT present (N = 8093)	
Sex			
Ambiguous or unknown	262 (0.1)	43 (0.5)	<.001
Girls	188 459 (46.9)	2646 (32.7)	
Boys	212 890 (53.0)	5404 (66.8)	
Gestational age, wk			
Mean (SD)	30.1 (2.84)	29.4 (3.04)	<.001
Median (range)	31.0 (23.0-33.0)	30.0 (23.0-33.0)	
Birthweight, kg			
Mean (SD)	1.49 (0.53)	1.38 (0.58)	<.001
Median (range)	1.52 (0.25-6.00)	1.32 (0.29-5.29)	
Race			
Asian	11 299 (2.8)	266 (3.3)	<.001
Black	92 093 (22.9)	1347 (16.6)	
Hispanic	77 596 (19.3)	1984 (24.5)	
White	190 394 (47.4)	3838 (47.4)	
Other	30 229 (7.5)	658 (8.1)	
Known genetic disorder			
Absent	398 563 (99.2)	7744 (95.7)	<.001
Present	3048 (0.8)	349 (4.3)	
Extrarenal			
Absent	381 829 (95.1)	6516 (80.5)	<.001
Present	19 782 (4.9)	1577 (19.5)	

Abbreviation: CAKUT, congenital anomalies of the kidney and urinary tract.

**Figure 1.**
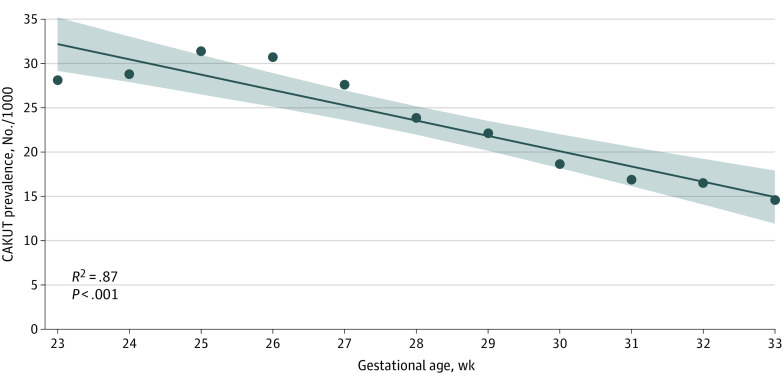
Prevalence of CAKUT by Gestational Age The prevalence of congenital anomalies of the kidneys and urinary tract (CAKUT), with the shaded area showing 95% CIs, was significantly correlated with prematurity, with higher prevalence at lower gestational ages.

Multiple logistic regression demonstrated a lower risk of CAKUT in infants born to mothers identifying as Black and higher risk in infants born to mothers identifying as Hispanic. A diagnosis of CAKUT was significantly associated with ambiguous or male sex, earlier gestational age, lower birthweight, the presence of known genetic disorders, and with congenital anomalies of other organ systems (eFigure 2 in the Supplement). There was an inverse linear correlation between the prevalence of CAKUT and earlier gestational age (*R^2^* = 0.87) (Figure 1). This association was mostly driven by UTD, which accounted for most anomalies (eTable 1 in the Supplement). We observed variable association between gestational age and CAKUT subcategories: gestational age was positively correlated with and RA, RHD, and multiple anomalies, and negatively correlated with UTD and not specified anomalies (eFigure 3 in the Supplement). Genetic disorders including aneuploidy, CNVs, and single-gene disorders were significantly more prevalent in infants with CAKUT (eTable 4 in the Supplement).

**Figure 2.**
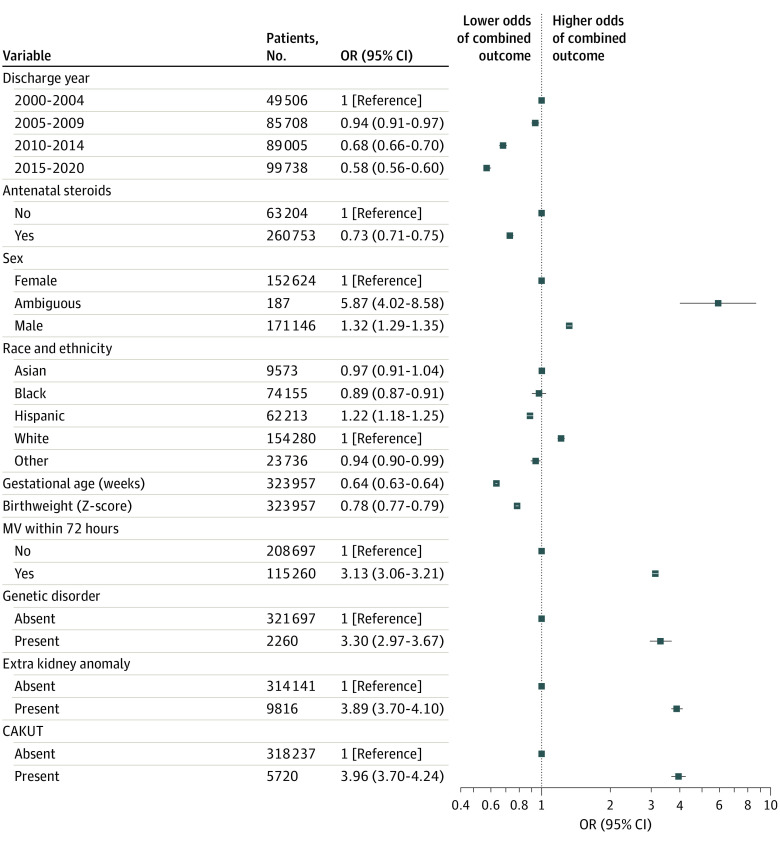
Multiple Logistic Regression for Death or Severe Illness Given Individual Characteristics Following Exclusion of Infants Transferred Prior to Reaching a Clinical End Point The presence of congenital anomalies of the kidneys and urinary tract (CAKUT) was associated with significantly higher odds of disease in preterm infants following adjustment for known risk factors. MV indicates mechanical ventilation; and OR, odds ratio.

After exclusion of infants transferred from or to other facilities, 323 957 infants were available for the study of clinical outcomes. Multiple logistic regression demonstrated that the presence of CAKUT was associated with a significantly higher OR (3.96, 95% CI, 3.70-4.24) of the combined outcome of death or severe illness when adjusted for known risk factors (Figure 2). Subgroup analyses found that the association between CAKUT and clinical outcomes was not driven by risk of individual severe illness. Rather, infants with CAKUT (isolated or with extrarenal anomalies) had significantly greater risk of every illness studied (Table 2; eFigure 4 in the Supplement). Likewise, subgroup analyses found the association was not driven by one form of CAKUT. Infants with all categories of CAKUT had higher rates of severe illness or death (eFigure 5 in the Supplement). The crude OR of severe illnesses or death in preterm infants with CAKUT was comparable with that found in infants with congenital anomalies of the heart and central nervous system (eFigure 6 in the Supplement).

**Table 2.** Odds of Death or Severe Illness in Preterm Infants With CAKUT

Outcome	OR (95% CI)	
	Isolated CAKUT	CAKUT with extrarenal features
Death or any severe illness	4.11 (3.88-4.35)	7.69 (6.66-8.87)
Death	2.01 (1.83-2.22)	7.05 (6.05-8.23)
AKI	5.71 (5.24-6.23)	9.13 (7.68-10.87)
Kidney failure	66.98 (34.50-130.06)	196.24 (87.22-441.50)
ICH	2.22 (2-2.47)	2.47 (1.94-3.14)
NEC	2.37 (2.13-2.65)	3.06 (2.42-3.88)
BPD	5.79 (5.11-6.57)	13.18 (10.69-16.27)
ROP	4.50 (3.93-5.16)	2.95 (2.00-4.33)
Sepsis	4.67 (4.39-4.97)	3.84 (3.29-4.48)
Shock	2.53 (2.35-2.71)	4.38 (3.78-5.08)

Abbreviations: AKI, acute kidney injury; BPD, bronchopulmonary dysplasia; CAKUT, congenital anomalies of the kidney and urinary tract; ICH, intracranial hemorrhage; NEC, necrotizing enterocolitis; OR, odds ratio; ROP, retinopathy of prematurity.

Repeating this analysis in a mixed effect model with cases nested by facility demonstrated the same associations, but with smaller effect sizes (eTables 3 and 4 in the Supplement). The rate of severe illness or death was compared across gestational ages in infants with no congenital anomalies, isolated CAKUT, and with CAKUT and additional extrarenal anomalies (Table 3; eFigure 7 in the Supplement). The presence of CAKUT was associated with significantly higher risk of death or severe illness after 24 and 27 weeks of gestation for infants with isolated CAKUT and CAKUT with extrarenal anomalies respectively. Additionally, the presence of CAKUT with extrarenal anomalies was associated with significantly higher risk compared to isolated CAKUT for infants born at 28 and 30-to-33-weeks’ gestation.

**Table 3.** Rates of Death or Severe Illness in Infants With and Without CAKUT

Gestational age, wk	Patients experiencing death or severe illness, No. (%)		
	No anomaly	Isolated CAKUT	CAKUT with extrarenal features
23	4800 (94)	120 (93)	13 (100)
24	7763 (86)	248 (96)^a^	23 (100)
25	7799 (74)	290 (88)^a^	29 (87)
26	7388 (60)	289 (83)^a^	29 (80)
27	6960 (45)	287 (74)^a^	33 (75)^a^
28	6579 (34)	254 (63)^a^	53 (85)^a^^,^^b^
29	5463 (24)	241 (59)^a^	49 (68)^a^^,^^b^
30	4769 (16)	189 (42)^a^	71 (73)^a^^,^^b^
31	4057 (10)	199 (37)^a^	60 (62)^a^^,^^b^
32	3765 (6)	211 (28)^a^	96 (58)^a^^,^^b^
33	2988 (3)	167 (18)^a^	99 (49)^a^^,^^b^

Abbreviation: CAKUT, congenital anomalies of the kidney and urinary tract.

^a^ *P* < .05 for Bonferroni-adjusted χ^2^ test compared to rate in infants without anomalies.

^b^ *P* < .05 for Bonferroni-adjusted χ^2^ test compared to rate in infants with isolated CAKUT.

## Discussion

To our knowledge, this represents the largest study of the prevalence and clinical associations of CAKUT in preterm infants. Our data showed CAKUT to be prevalent in preterm infants, more than has been described in the general population. The prevalence of CAKUT, particularly UTD anomalies, was associated with earlier gestational age. Interestingly, extrapolating this association to term gestation of 37 to 40 weeks projects to a prevalence of 30 to 80 cases per 10 000 individuals, similar to rates found in general population studies.^9,10,11,12,13,14,15,16,17^ We show that every CAKUT subcategory is independently associated with higher mortality, with odds ratios exceeding those for traditional risk factors such as mechanical ventilation.

Interpretation of the prevalence of UTD is challenging in part because of variable diagnostic terms (eg, hydronephrosis, pelviectasis, pelvic fullness) and diagnostic criteria (eg, size of renal pelvis, dilation of calyces, changes in kidney parenchyma).^26,27^ Furthermore it is unknown at what gestational or postnatal age anomalies were diagnosed. A higher frequency of ultrasounds in pregnancies at risk of preterm delivery may account for higher detection of UTD. Nevertheless, our data suggest that UTD should be considered as a morbid phenotype for preterm infants, and future studies should investigate the association of specific UTD subcategories and outcomes in premature infants. Notably, our data did not include descriptions of the severity of UTD anomalies. Mild UTD may have a lower association with death or severe illness.

We found that CAKUT was associated with genetic disorders and extrarenal anomalies. CAKUT has been previously shown to have a strong genetic underpinning, with recent research identifying single-gene or CNV disorders responsible for 10% to 25% of cases.^4,5,6,7,28^ The majority of the genetic diagnoses in this study were aneuploidies and CNV disorders, which are associated with anomalies of multiple organs including the urinary tract. The association of genetic disorders with mortality in preterm infants extends recent data linking CNV disorders with mortality in adults in the UK Biobank, indicating a high health burden conferred across the lifespan.^29^ Thus, rather than CAKUT being an isolated finding, it may signify the sentinel feature of an underlying genetic disorder that contributes to severe illness. We note that genetic diseases were likely underestimated in this study since patients were not systematically tested for genetic disorders by sequencing approaches. Consistent with this hypothesis, genetic disorders and extrarenal anomalies each independently contributed to infant mortality in this study, suggesting unaccounted-for genetic disorders or developmental factors that contributed to adverse outcome.

## Strengths and Limitations

Limitations of this study included detection bias for urinary tract anomalies in high-risk pregnancies or in infants with critical illness. Furthermore, electronic health records lacked detail regarding timing of diagnoses or description of anomalies.

Despite these limitations, our study is to our knowledge the largest-to-date of CAKUT in preterm infants. We analyzed over 400 000 infants, representing 20% of infants born in the US during the study period. CAKUT was prevalent in preterm infants and associated with severe illness and death. The association may indicate the direct deleterious consequences of kidney and urinary malformations but may also reflect an underlying burden of genetic or developmental disorders, or environmental exposures that predispose to illness and death.

## Conclusions

These findings have important potential implications for clinical practice and research. First, the presence of CAKUT should prompt clinicians to consider a genetic workup. We demonstrated an association between genetic disorders including aneuploidy, CNVs, and single-gene disorders. This is consistent with the recent guidelines of the American College of Medical Genetics and Genomics, which recommend genome-wide sequencing for individuals with congenital anomalies and developmental disorders.^30^ Second, the presence of CAKUT may be considered as a risk factor for serious morbidity and mortality. The care of preterm infants often necessitates risk-benefit consideration in planning care. Our findings may prompt higher pretest probability for sepsis, shock, AKI, NEC, and other serious illnesses encountered frequently in neonatal practice. And this association may influence counseling and discussions with families, particularly in high-risk cases. Finally, these findings indicate the need for multiple lines of research. Prospective screening and longitudinal surveillance of preterm infants would clarify the risk of death or severe illness imparted by kidney anomalies. Prospective genome-wide sequencing of preterm infants with CAKUT would delineate the genetic architecture of this disease, identify specific gene-disease associations, and inform improved clinical management.

## Notes

Supplement. **eMethods.** R script used for data analysis **eTable 1.** Prevalence of CAKUT by gestational age and subdivided by CAKUT category **eTable 2.** Summary of multiple logistic regression for death or severe illness given individual characteristics **eTable 3.** Summary of multiple logistic regression for death or severe illness given individual characteristics as mixed effect model with cases nested by NICU facility **eTable 4.** Genetic disorders in infants with and without CAKUT **eFigure 1.** Prevalence of CAKUT in this cohort and in published studies of the general population **eFigure 2.** Multiple logistic regression for presence of CAKUT given individual characteristics **eFigure 3.** Correlation between gestational age and prevalence of CAKUT **eFigure 4.** Odds of severe illness in preterm infants with CAKUT **eFigure 5.** Odds of death or severe illness in preterm infants with CAKUT stratified by CAKUT categorization **eFigure 6.** Odds of death or severe illness in preterm infants with CAKUT and other congenital anomalies **eFigure 7.** Rates of death or severe illness by gestational age in infants with and without CAKUT
