# Supplementary material for: The Prevalence and Clinical Significance of Congenital Anomalies of the Kidney and Urinary Tract in Preterm Infants
Source: JAMA Netw Open. 2022 Sep 14;5(9):e2231626. doi: 10.1001/jamanetworkopen.2022.31626 (PMC9475384; doi:10.1001/jamanetworkopen.2022.31626)
Supplement: Supplement. — eMethods. R script used for data analysis eTable 1. Prevalence of CAKUT by gestational age and subdivided by CAKUT category eTable 2. Summary of multiple logistic regression for death or severe illness given individual characteristics eTable 3. Summary of multiple logistic regression for death or severe illness given individual characteristics as mixed effect model with cases nested by NICU facility eTable 4. Genetic disorders in infants with and without CAKUT eFigure 1. Prevalence of CAKUT in this cohort and in published studies of the general population eFigure 2. Multiple logistic regression for presence of CAKUT given individual characteristics eFigure 3. Correlation between gestational age and prevalence of CAKUT eFigure 4. Odds of severe illness in preterm infants with CAKUT eFigure 5. Odds of death or severe illness in preterm infants with CAKUT stratified by CAKUT categorization eFigure 6. Odds of death or severe illness in preterm infants with CAKUT and other congenital anomalies eFigure 7. Rates of death or severe illness by gestational age in infants with and without CAKUT [file jamanetwopen-e2231626-s001.pdf]

Supplemental Online Content

Hays T, Thompson MV, Bateman DA, et al. The prevalence and clinical significance of congenital anomalies of the kidney and urinary tract in preterm infants. *JAMA Netw Open.* 2022;5(9):e2231626. doi:10.1001/jamanetworkopen.2022.31626

eMethods. R script used for data analysis

eTable 1. Prevalence of CAKUT by gestational age and subdivided by CAKUT category

eTable 2. Summary of multiple logistic regression for death or severe illness given individual characteristics

eTable 3. Summary of multiple logistic regression for death or severe illness given individual characteristics as mixed effect model with cases nested by NICU facility

eTable 4. Genetic disorders in infants with and without CAKUT

eFigure 1. Prevalence of CAKUT in this cohort and in published studies of the general population

eFigure 2. Multiple logistic regression for presence of CAKUT given individual characteristics

eFigure 3. Correlation between gestational age and prevalence of CAKUT

eFigure 4. Odds of severe illness in preterm infants with CAKUT

eFigure 5. Odds of death or severe illness in preterm infants with CAKUT stratified by CAKUT categorization

eFigure 6. Odds of death or severe illness in preterm infants with CAKUT and other congenital anomalies

eFigure 7. Rates of death or severe illness by gestational age in infants with and without CAKUT

This supplemental material has been provided by the authors to give readers additional information about their work.

© 2022 Hays T et al. *JAMA Network Open.*

eMethods

The following script was used to generate tables and figures. The script was created using RStudio (version 2022.02.0).

```
#### Table 1.

library(table1)

## Read and label data

Prevalence <- read.csv("Prevalence8.csv")

label(Prevalence$BW) <- "Birthweight (kg)"
label(Prevalence$GA) <- "Gestational Age (weeks)"
label(Prevalence$Genetic) <- "Known Genetic Disorder"
label(Prevalence$Any_Extrarenal) <- "Extrarenal Anomaly"

## Calculate p-values for Table1

pvalue <- function(x, ...) {
  # Construct vectors of data y, and groups (strata) g
  y <- unlist(x)
  g <- factor(rep(1:length(x), times=sapply(x, length)))
  if (is.numeric(y)) {
    # For numeric variables, perform a standard 2-sample t-test
    p <- t.test(y ~ g)$p.value
  } else {
    # For categorical variables, perform a chi-squared test of independence
    p <- chisq.test(table(y, g))$p.value
  }
  # Format the p-value, using an HTML entity for the less-than sign.
  # The initial empty string places the output on the line below the variable label.
  c("", sub("<", "&lt;", format.pval(p, digits=1, eps = 1e-100)))
}

##  Table 1.

table1(~Sex + GA + BW + Race + Genetic + Extrarenal | CAKUT, data=Prevalence,
overall=F, extra.col=list(`P-value`=pvalue))


#### Figure 1 and eTable1

## Figure 1
```

© 2022 Hays T et al. *JAMA Network Open.*

```
library(ggpubr)
library(ggplot2)
library(ggpmisc)

Simp_Reg <- read.csv("GA_CAKUT_Simple_Reg.csv")

## Simple liner regression

LinReg1 <- lm(CAKUT ~ GA, data = Simp_Reg)
summary(LinReg1)

## Graph of regression model

Any_CAKUT <- ggplot(LinReg1, aes(x = GA, y = CAKUT)) +
  geom_point() +
  stat_smooth(method = lm) +
  theme_classic() +
  labs(x = "Gestatational Age (weeks)", y = "CAKUT Prevalence per 1,000") +
  scale_x_continuous(breaks = seq(23, 33, by = 1)) +
  stat_regline_equation(label.y.npc = 0.85, label.x.npc = 0.67, size = 6, aes(label =
..rr.label..))+
  annotate("text", x=31.02, y = 27, size = 6, label = "P-value < 0.001")

Any_CAKUT

## ETable 1

library(tidyverse)
library(pixiedust)
library(kableExtra)
library(webshot)

Pos <-read.csv("CAKUTbyGA.csv")

tbl1 <- dust(Pos)

tbl1 %>%
  sprinkle_colnames(GA = "Gestational Age (weeks)") %>%
  kable() %>% kable_styling() %>%
  add_header_above(c(" " = 1, "No. Affected by CAKUT (prevalence per 1,000)*" =
7))%>%
  kable_classic(full_width = F, html_font = "Arial") %>%
  footnote("AWD: abdominal wall defects, EF: ectopy or fusion, RA: unilateral or bilateral
agenesis, RHD: renal hypoplasia or dysplasia, UTD: urinary tract dilation and anomalies
of the urethra or ureters Mul: multiple different forms of CAKUT, NOS: forms of CAKUT
not otherwise specified", general_title = "*Abbreviations")
```

© 2022 Hays T et al. *JAMA Network Open.*

```
#### Figure 2 and eTables 3 and 3

library(expss)
library(gtsummary)
library(forestmodel)
library(lme4)
library(lmerTest)

## Data preparation

CAKUT_MM<- read.csv("MMModel4.csv")

CAKUT_MM$DischargeYear <- factor(CAKUT_MM$DischargeYear)
CAKUT_MM$Sex <- factor(CAKUT_MM$Sex)
CAKUT_MM$Race <- factor(CAKUT_MM$Race)
CAKUT_MM$AntenatalSteroids <- factor(CAKUT_MM$AntenatalSteroids)
CAKUT_MM$VENTD02 <- factor(CAKUT_MM$VENTD02)
CAKUT_MM$Genetic <- factor(CAKUT_MM$Genetic)
CAKUT_MM$AnyExtraRenal <- factor(CAKUT_MM$AnyExtraRenal)
CAKUT_MM$AnyCAKUT <- factor(CAKUT_MM$AnyCAKUT)

CAKUT_MM$DischargeYear <- relevel(CAKUT_MM$DischargeYear, ref = "2000 to
2004")
CAKUT_MM$Sex <- relevel(CAKUT_MM$Sex, ref = "Female")
CAKUT_MM$Race <- relevel(CAKUT_MM$Race, ref = "White")

CAKUT_MM <- apply_labels(CAKUT_MM, DischargeYear = "Discharge Year",
AntenatalSteroids = "Antenatal Steroids", GA = "Gestational Age (weeks)", BW_Z_TH =
"Birthweight (Z-score)", VENTD02 = "MV within 72 hours", AnyCAKUT = "CAKUT",
Genetic = "Genetic Disorder", AnyExtraRenal = "Extrarenal Anomaly")

## multiple variable logistic regression model

LogReg <- glm(CritIllorDeath ~ DischargeYear + AntenatalSteroids + Sex + Race + GA
+ BW_Z_TH + VENTD02 + Genetic + AnyExtraRenal + AnyCAKUT , data =
CAKUT_MM, family = "binomial")

summary(LogReg, digits = 1000)

plot(density(resid(LogReg, type='pearson')))
plot(residuals(LogReg))

## Table depiction of model
```

© 2022 Hays T et al. *JAMA Network Open.*

```
tbl1 <- tbl_regression(LogReg, exponentiate = TRUE, pvalue_fun = ~style_pvalue(.x,
digits = 3))
tbl1 <- modify_header(tbl1, estimate ="**OR**") %>%  modify_footnote(estimate = "OR
= Odds Ratio", abbreviation = TRUE)
tbl1


## Graph of model

# Preparation of panels and formatting of p-value

panels <- list(
  list(width = 0.03),
  list(width = 0.1, display = ~variable, fontface = "bold", heading = "Variable"),
  list(width = 0, display = ~level, heading = ""),
  list(width = 0.05, display = ~n, hjust = 1, heading = "Patients, No."),
  list(width = 0.03, hjust = 0.5),
  list(width = 0.12, display = ~ ifelse(reference, "Reference", sprintf(
    "%0.2f (%0.2f, %0.2f)",
    trans(estimate), trans(conf.low), trans(conf.high)
  )), display_na = NA, heading = "OR (95% CI)"),
  list(width = 0.03, item = "vline", hjust = 0.5),
  list(width = 0.55, item = "forest", hjust = 0.5, heading = "", linetype = "dashed",
     line_x = 0),
  list(width = 0.03))

forest_model(LogReg, panels, factor_separate_line = TRUE)


## Model as mixed effect model with each individual nested within healthcare facility

CAKUT_MM <- apply_labels(CAKUT_MM, DischargeYear = "Discharge Year",
AntenatalSteroids = "Antental Steroids", GA = "Gestational Age (weeks)", BW_Z_TH =
"Birthweight (Z-score)", VENTD02 = "MV within 72 hours", AnyCAKUT = "CAKUT",
Genetic = "Genetic Disorder", AnyExtraRenal = "Extrarenal Anomaly")

model <- lmer(CritIllorDeath ~ DischargeYear + AntenatalSteroids + Sex + Race + GA +
BW_Z_TH + VENTD02 + Genetic + AnyExtraRenal + AnyCAKUT +
(1|Facility_Code/PatientRandomSeqID),
              data = CAKUT_MM,
              control=lmerControl(check.nobs.vs.nlev = "ignore",
                                  check.nobs.vs.rankZ = "ignore",
                                  check.nobs.vs.nRE="ignore",  optimizer = "Nelder_Mead"))
summary(model, digits = 1000)
plot(density(resid(model, type='pearson')))
```

© 2022 Hays T et al. *JAMA Network Open.*

```
plot(residuals(model))

tbl2 <-  tbl_regression(model, exponentiate = TRUE, pvalue_fun = ~style_pvalue(.x,
digits = 3))
tbl2 <- modify_header(tbl2, estimate ="**OR**") %>%  modify_footnote(estimate = "OR
= Odds Ratio", abbreviation = TRUE)
tbl2


#### Table 2 and eFigure 4

## Table 2
library(tidyverse)
library(pixiedust)
library(kableExtra)
library(webshot)

Pos <-read.csv("Odds_MM_CAKUT_Iso_Syn.csv")

tbl1 <- dust(Pos)

tbl1 %>%
  sprinkle_colnames(Outcome = "Outcome*", Isolated = "Isolated CAKUT", Syndromic=
"CAKUT with Extrarenal Features") %>%
  kable() %>% kable_styling() %>%
  add_header_above(c(" " = 1, "OR (95% CI)" = 2))%>%
  kable_classic(full_width = F, html_font = "Arial") %>%
  footnote("AKI: acute kidney injury, ICH: intracranial hemorrhage, NEC: necrotizing
enterocolitis, BPD: bronchopulmonary dysplasia, ROP: retinopathy of prematurity",
general_title = "*Abbreviations")

##e Figure 4

library(ggplot2)

# Create labels
boxLabels = c("Death or Any Severe Illness", "Death", "AKI", "Renal Failure", "IVH or
PVL","NEC or SIP", "Severe BPD", "Severe ROP", "Culutre-positive Sepsis", "Shock
Requiring Pressor")

# Entering summarized data of ORs and CIs of isolated CAKUT
df1 <- data.frame(yAxis = length(boxLabels):1,
                boxOdds = c(4.12, 2.02, 5.72, 66.99, 2.22, 2.38, 5.8, 4.51, 4.68, 2.53),
                boxCILow = c(3.89, 1.83, 5.24, 34.50, 2.0, 2.13, 5.12, 3.94, 4.4, 2.36),
                boxCIHigh = c(4.36, 2.23, 6.23, 130.06, 2.47, 2.66, 6.58, 5.16, 4.98, 2.72))
```

© 2022 Hays T et al. *JAMA Network Open.*

```
df1$group <- "Isolated CAKUT"

# Entering summarized data of ORs and CIs of syndromic CAKUT
df2 <- data.frame(yAxis = length(boxLabels):1,
                 boxOdds = c(7.69, 7.06, 9.14, 196.24, 2.47, 3.07, 13.19, 2.95, 3.84, 4.39),
                 boxCILow = c(6.66, 6.05, 7.68, 87.23, 1.94, 2.42, 10.69, 2.01, 3.3, 3.78),
                 boxCIHigh = c(8.88, 8.23, 10.87, 441.51, 3.15, 3.89, 16.27, 4.34, 4.49, 5.09))

df2$group <- "CAKUT with Extrarenal Anomaly"

df3 = rbind(df1,df2)


# Plot



p <- ggplot(df3, aes(x = boxOdds, y = yAxis, color=group))

p + geom_vline(aes(xintercept = 1), size = .25, linetype = "dashed") +
  geom_errorbarh(aes(xmax = boxCIHigh, xmin = boxCILow), size = .5, height = .2,
position=ggstance::position_dodgev(height=-0.5)) +
  geom_point(shape = 15, size = 3.5, position=ggstance::position_dodgev(height=-0.5))
+
  theme_classic() +
  labs(color = "") +
  theme(legend.position = c(0.8, 0.2)) +
  theme(panel.grid.minor = element_blank()) +
  scale_y_continuous(breaks = seq(1,10,1), labels = c("Shock", "Sepsis", "ROP", "BPD",
"NEC", "ICH","Renal Failure", "AKI", "Death", "Death or Any Severe Illness")) +
  ylab("") +
  xlab("Odds Ratio (95% Confidence Interval; Log Scale)") +
  theme(text = element_text(size = 15)) +
  coord_trans(x = 'log10') +
  scale_x_continuous(breaks = c(1,2.5,5, 7.5,10,100),
                     minor_breaks = NULL,
                     labels = c(1,2.5,5, 7.5,10,100))


#### Table 3 and eFigure 7

## Table 3

library(tidyverse)
library(pixiedust)
library(kableExtra)
```

© 2022 Hays T et al. *JAMA Network Open.*

```
library(webshot)

Pos <-read.csv("Outcomes_GA.csv")

tbl1 <- dust(Pos)

tbl1 %>%
  sprinkle_colnames(GA = "Gestational Age (weeks)", None= "No Anomaly", Isolated =
"Isolated CAKUT", Syndromic= "CAKUT with Extrarenal Features") %>%
  kable() %>% kable_styling() %>%
  add_header_above(c(" " = 1, "No. Experiencing Death or Severe Illness (%)" =
3))%>%
  kable_classic(full_width = F, html_font = "Arial") %>%
  footnote("AKI: acute kidney injury, ICH: intracranial hemorrhage, NEC: necrotizing
enterocolitis, BPD: bronchopulmonary dysplasia, ROP: retinopathy of prematurity",
general_title = "*Abbreviations")


## eFigure 7

library(geomsignif)
library(rstatix)

## Entry of number of infants at each gestational age, and their rate or death or severe
illness

Data1 <- structure(list(Condition = structure(c(1L, 1L, 1L, 1L, 1L, 1L, 1L, 1L, 1L, 1L, 1L,
                                           2L, 2L, 2L, 2L, 2L, 2L, 2L, 2L, 2L, 2L, 2L,
                                           3L, 3L, 3L, 3L, 3L, 3L, 3L, 3L, 3L, 3L, 3L),
                                         .Label = c("No Anomaly", "Isolated CAKUT",
                                                    "CAKUT with Extrarenal Anomaly"), class =
"factor"),
                   Electrode = structure(c(1L, 2L, 3L, 4L, 5L, 6L, 7L, 8L, 9L, 10L, 11L,
                                           1L, 2L, 3L, 4L, 5L, 6L, 7L, 8L, 9L, 10L, 11L,
                                           1L, 2L, 3L, 4L, 5L, 6L, 7L, 8L, 9L, 10L, 11L),
                                         .Label = c("23", "24", "25", "26", "27", "28", "29", "30",
"31","32", "33"), class = "factor"),
                   N =
c(5075,8957,10479,12272,15195,19170,22204,29341,38889,60873,85229,128,258,327
,348,385,401,403,442,533,733,923,13,23,33,36,44,62,72,96,96,163,201),
                   measurement =
c(0.94581,0.86670,0.74425,0.60202,0.45805,0.34319,0.24604,0.16254,0.10458,0.0618
5,0.03506,0.93750,0.96124,0.88685,0.83046,0.74545,0.63342,0.59801,0.42760,0.3733
6,0.28786,0.18093,1.00000,1.00000,0.87879,0.80556,0.75000,0.85484,0.68056,0.7395
8,0.62500,0.58896,0.49254)),
               row.names = c(NA, -33L), class = "data.frame")
```

© 2022 Hays T et al. *JAMA Network Open.*

```
## Creation of plot of rate of illness by GA, leaving extra room above for p-value bars

p4 <- ggplot(Data1, aes(fill=Condition, x = Electrode, y = measurement)) +
  geom_bar(stat = "identity", position = "dodge")  +
scale_y_continuous(breaks=c(0,0.25,0.5,0.75,1),limits=c(0,1.1)) +
  theme(axis.text.x = element_text(size = 16,face="bold"),
        axis.text.y = element_text(size = 16, face = "bold"),
        axis.title = element_text(face = "bold")) + xlab("Gestational Age (weeks)") +
ylab("Rate of Severe Illness or Death") +
  theme_classic()+
  scale_fill_brewer(palette="Blues")+
  labs(fill = "")


p4

## chi sq tests by GA with Bonferroni correction and pairwise p-values

GA_Death <-read.csv("GAxCAKUT_Outcome_Ill_OR_Dead2.csv")
GA_Death <-read.csv("Outcome.csv")

GA_Death$GA <- factor(GA_Death$GA)

Outcome_rate <- GA_Death %>%
  group_by(GA, Anomaly) %>%
  mutate(percent = Number/(sum(Number))) %>%
  filter(Outcome == "Death or Severe Illness")


xtab23 <- subset(GA_Death, GA == "23")
freq_table23 <-table(xtab23$Anomaly, xtab23$Outcome)
pairwise.prop.test(freq_table23, p.adjust ="bonferroni")


xtab24 <- subset(GA_Death, GA == "24")
freq_table24 <-table(xtab24$Anomaly, xtab24$Outcome)
pairwise.prop.test(freq_table24, p.adjust ="bonferroni")

xtab25 <- subset(GA_Death, GA == "25")
freq_table25 <-table(xtab25$Anomaly, xtab25$Outcome)
pairwise_prop_test(freq_table25, p.adjust.method = "bonferroni")

xtab26 <- subset(GA_Death, GA == "26")
freq_table26 <-table(xtab26$Anomaly, xtab26$Outcome)
pairwise_prop_test(freq_table26, p.adjust.method = "bonferroni")
```

© 2022 Hays T et al. *JAMA Network Open.*

```
xtab27 <- subset(GA_Death, GA == "27")
freq_table27 <-table(xtab27$Anomaly, xtab27$Outcome)
pairwise_prop_test(freq_table27, p.adjust.method = "bonferroni")

xtab28 <- subset(GA_Death, GA == "28")
freq_table28 <-table(xtab28$Anomaly, xtab28$Outcome)
pairwise_prop_test(freq_table28, p.adjust.method = "bonferroni")

xtab29 <- subset(GA_Death, GA == "29")
freq_table29 <-table(xtab29$Anomaly, xtab29$Outcome)
pairwise_prop_test(freq_table29, p.adjust.method = "bonferroni")

xtab30 <- subset(GA_Death, GA == "30")
freq_table30 <-table(xtab30$Anomaly, xtab30$Outcome)
pairwise_prop_test(freq_table30, p.adjust.method = "bonferroni")

xtab31 <- subset(GA_Death, GA == "31")
freq_table31 <-table(xtab31$Anomaly, xtab31$Outcome)
pairwise_prop_test(freq_table31, p.adjust.method = "bonferroni")

xtab32 <- subset(GA_Death, GA == "32")
freq_table32 <-table(xtab32$Anomaly, xtab32$Outcome)
pairwise_prop_test(freq_table32, p.adjust.method = "bonferroni")

xtab33 <- subset(GA_Death, GA == "33")
freq_table33 <-table(xtab33$Anomaly, xtab33$Outcome)
pairwise_prop_test(freq_table33, p.adjust.method = "bonferroni")


## creation of p-value brackets

p4 + geom_signif(
  y_position = c(1.1, 1.1,
                 1.1,1.1,1.1,
                 1.1,1.1, 1.1,
                 1.1,1.1),
  xmin = c(1.8, 2.8,
           3.8,4.8, 5.8,
           6.8, 7.8, 8.8,
           9.8, 10.8),
  xmax = c(2.2, 3.2,
           4.2,5.2, 6.2,
           7.2,8.2, 9.2,
           10.2, 11.2),
  annotation = c("a", "a",
```

© 2022 Hays T et al. *JAMA Network Open.*

```
                "a","a, b", "a, b, c",
                "a, b","a, b, c", "a, b, c",
                "a, b, c", "a, b, c"),
  tip_length = 0.01, textsize = 3, size = 0.3)



#### Supplemental Figure 6
library(ggplot2)

boxLabels = c("Any CAKUT", "AWD", "EF", "RA", "RHD", "UTD", "NOS", "Mul")

# Entering summarized data of ORs and CIs of isolated CAKUT

df_I <- data.frame(yAxis = length(boxLabels):1,
                boxOdds = c(3.88, 5.64, 1.56, 2.30, 3.30, 4.20, 2.88, 5.51),
                boxCILow = c(3.67, 2.19, 0.98, 1.59, 2.63, 3.93, 2.49, 3.78),
                boxCIHigh = c(4.11, 14.56, 2.47, 3.16, 4.13, 4.50, 3.34, 8.02))

df_I$group <- "Isolated CAKUT"

# Entering summarized data of ORs and CIs of syndromic CAKUT
df_S <- data.frame(yAxis = length(boxLabels):1,
                boxOdds = c(7.26, 7.90, 4.67, 5.99, 12.72, 6.82, 5.58, 19.16),
                boxCILow = c(6.29, 2.74, 2.05, 3.34, 6.87, 5.71, 3.87, 8.01),
                boxCIHigh = c(8.37, 22.74, 10.65, 10.74, 23.53, 8.16, 8.05, 45.82))

df_S$group <- "CAKUT with Extrarenal Anomaly"

df_Combo = rbind(df_I,df_S)

# Plot

p <- ggplot(df_Combo, aes(x = boxOdds, y = yAxis, color=group))

p + geom_vline(aes(xintercept = 1), size = .25, linetype = "dashed") +
  geom_errorbarh(aes(xmax = boxCIHigh, xmin = boxCILow), size = .5, height = .2,
position=ggstance::position_dodgev(height=-0.5)) +
  geom_point(shape = 15, size = 3.5, position=ggstance::position_dodgev(height=-0.5))
+
  theme_classic() +
  labs(color = "") +
  theme(legend.position = c(0.8, 0.3)) +
  theme(panel.grid.minor = element_blank()) +
  scale_y_continuous(breaks = seq(1,8,1), labels = c("Mul", "NOS", "UTD", "RHD", "RA",
"EF", "AWD", "Any CAKUT")) +
```

© 2022 Hays T et al. *JAMA Network Open.*

```
  ylab("") +
  xlab("Odds Ratio (95% Confidence Interval; Log Scale)") +
  theme(text = element_text(size = 15)) +
  coord_trans(x = 'log10') +
  scale_x_continuous(breaks = c(1,2.5,5, 7.5,10,100),
                     minor_breaks = NULL,
                     labels = c(1,2.5,5, 7.5,10,100))


#### e Figure 1

Prev_Comps <-read.csv("Prevalence_Comparisons3.csv")

Prev_Comps$CAKUT <- factor(Prev_Comps$CAKUT, levels = c("AWD", "EF", "RA",
"RHD", "UTD", "NOS", "Mul"))

Prev_Comps_Bars <- ggplot(Prev_Comps, aes(fill=CAKUT, y=Prevalence, x=Cohort)) +
geom_bar(position="stack", stat="identity") +
  theme_classic()+
  scale_fill_brewer(palette="RdBu") +
  labs(x = "Cohort", y = "CAKUT Prevalence (per 10,000)") +
  theme(legend.position = c(0.8, 0.8)) +
  labs(fill = "")

Prev_Comps_Bars


#### e Figure 2

library(expss)
library(gtsummary)
library(forestmodel)

## Data Entry

Prevalence3 <- read.csv("Prevalence8.csv")

Prevalence3$CAKUT <-factor(Prevalence3$CAKUT)
Prevalence3$Race <- factor(Prevalence3$Race)
Prevalence3$Sex <- factor(Prevalence3$Sex)

Prevalence3$Sex <- relevel(Prevalence3$Sex, ref = "Female")
Prevalence3$Race <- relevel(Prevalence3$Race, ref = "White")
```

© 2022 Hays T et al. *JAMA Network Open.*

```
Prevalence3 <- apply_labels(Prevalence3, GA = "Gestational Age (weeks)", Sex =
"Sex", BW_Z_TH = "Birthweight (Z score)", Genetic = "Genetic Disorder",
Extrarenal_Detail = "Extrarenal Anomaly")

## Multivariable logistic regression model

LogReg3 <- glm(CAKUT~ Sex + Race + GA + BW_Z_TH + Genetic + Extrarenal_Detail,
data = Prevalence3, family = "binomial")

plot(residuals(LogReg3))
hist(residuals(LogReg3), main = "Histogram of Residuals", xlab = "residuals")

tbl2 <- tbl_regression(LogReg3, exponentiate = FALSE)
tbl2 <- modify_header(tbl2, estimate = "**Beta**") %>% modify_footnote(update =
everything() ~ NA, abbreviation = TRUE) %>% modify_footnote(ci = "Confidence
Interval", abbreviation = TRUE)
tbl2

## plotting of model

panels <- list(
  list(width = 0.03),
  list(width = 0.1, display = ~variable, fontface = "bold", heading = "Variable"),
  list(width = 0.1, display = ~level,fontface = "bold", heading = "Value"),
  list(width = 0.05, display = ~n, hjust = 1, heading = "No."),
  list(width = 0.03, hjust = 0.5),
  list(width = 0.12, display = ~ ifelse(reference, "Reference", sprintf(
    "%0.2f (%0.2f, %0.2f)",
    trans(estimate), trans(conf.low), trans(conf.high)
  )), display_na = NA, heading = "OR (95% CI)"),
  list(width = 0.03, item = "vline", hjust = 0.5),
  list(width = 0.55, item = "forest", hjust = 0.5, heading = "", linetype = "dashed",
       line_x = 0),
  list(width = 0.03))


forest_model(LogReg3, panels, factor_separate_line = TRUE)



##### e Figure 3

library(tidyverse)
library(ggpubr)
library(ggplot2)
library(ggpmisc)
```

© 2022 Hays T et al. *JAMA Network Open.*

```
## Simple Linear Regression for presence of any CAKUT by GA

Simp_Reg <- read.csv("GA_CAKUT_Simple_Reg.csv")


LinReg1 <- lm(CAKUT ~ GA, data = Simp_Reg)
summary(LinReg1)

Any_CAKUT <- ggplot(LinReg1, aes(x = GA, y = CAKUT)) +
  geom_point() +
  stat_smooth(method = lm) +
  labs(x = "Gestational Age (weeks)", y = "CAKUT Prevalence per 1,000") +
  scale_x_continuous(breaks = seq(23, 33, by = 1)) +
  stat_regline_equation(label.y.npc = 0.85, label.x.npc = 0.75, aes(label = ..rr.label..)) +
  annotate("text", x=24, y = 35, size = 7, label = "") +
  theme_classic()+
  annotate("text", x=31.02, y = 29, size = 4, label = "P-value < 0.001")

Any_CAKUT


## Simple Linear Regression for presence of AWD by GA

AWD_Reg <- read.csv("GA_AWD_Simple_Reg.csv")

LinReg2 <- lm(CAKUT ~ GA, data = AWD_Reg)

AWD <- ggplot(LinReg2, aes(x = GA, y = CAKUT)) +
  geom_point() +
  stat_smooth(method = lm) +
  labs(x = "Gestational Age (weeks)", y = "AWD Prevalence per 1,000") +
  scale_x_continuous(breaks = seq(23, 33, by = 1)) +
  stat_regline_equation(label.y.npc = 0.18, label.x.npc = 0.75, aes(label = ..rr.label..))+
  stat_fit_glance(aes(label = paste("P-value =", signif(..p.value.., digits = 2))),
label.x=0.98, label.y = 0.1)+
  theme_classic()


AWD

## Simple Linear Regression for presence of EF by GA

EF_Reg <- read.csv("GA_EF_Simple_Reg.csv")

LinReg3 <- lm(CAKUT ~ GA, data = EF_Reg)
```

© 2022 Hays T et al. *JAMA Network Open.*

```
EF <- ggplot(LinReg3, aes(x = GA, y = CAKUT)) +
  geom_point() +
  stat_smooth(method = lm) +
  labs(x = "Gestational Age (weeks)", y = "EF Prevalence per 1,000") +
  scale_x_continuous(breaks = seq(23, 33, by = 1)) +
  stat_regline_equation(label.y.npc = 0.75, label.x.npc = 0.75, aes(label = ..rr.label..))+
  stat_fit_glance(aes(label = paste("P-value =", signif(..p.value.., digits = 2))),
label.x=0.98, label.y = 0.7) +
  theme_classic()

EF


## Simple Linear Regression for presence of RA by GA

RA_Reg <- read.csv("GA_RA_Simple_Reg.csv")

LinReg4 <- lm(CAKUT ~ GA, data = RA_Reg)

RA <- ggplot(LinReg4, aes(x = GA, y = CAKUT)) +
  geom_point() +
  stat_smooth(method = lm) +
  labs(x = "Gestational Age (weeks)", y = "RA Prevalence per 1,000") +
  scale_x_continuous(breaks = seq(23, 33, by = 1)) +
  stat_regline_equation(label.y.npc = 0.2, label.x.npc = 0.74, aes(label = ..rr.label..))+
  theme_classic()+
  annotate("text", x=31.02, y = 0.1, size = 4, label = "P-value < 0.001")

RA


## Simple Linear Regression for presence of RHD by GA

RHD_Reg <- read.csv("GA_RHD_Simple_Reg.csv")

LinReg5 <- lm(CAKUT ~ GA, data = RHD_Reg)

RHD <- ggplot(LinReg5, aes(x = GA, y = CAKUT)) +
  geom_point() +
  stat_smooth(method = lm) +
  labs(x = "Gestational Age (weeks)", y = "RHD Prevalence per 1,000") +
  scale_x_continuous(breaks = seq(23, 33, by = 1)) +
  stat_regline_equation(label.y.npc = 0.09, label.x.npc = 0.75, aes(label = ..rr.label..))+
  stat_fit_glance(aes(label = paste("P-value =", signif(..p.value.., digits = 1))),
label.x=0.95, label.y = 0.1) +
```

© 2022 Hays T et al. *JAMA Network Open.*

```
  theme_classic()

RHD

## Simple Linear Regression for presence of UTD by GA

UTD_Reg <- read.csv("GA_UTD_Simple_Reg.csv")

LinReg6 <- lm(CAKUT ~ GA, data = UTD_Reg)

UTD <- ggplot(LinReg6, aes(x = GA, y = CAKUT)) +
  geom_point() +
  stat_smooth(method = lm) +
  labs(x = "Gestational Age (weeks)", y = "UTD Prevalence per 1,000") +
  scale_x_continuous(breaks = seq(23, 33, by = 1)) +
  stat_regline_equation(label.y.npc = 0.1, label.x.npc = 0.73, aes(label = ..rr.label..))+
  theme_classic() +
  annotate("text", x=31.02, y = 0.1, size = 4, label = "P-value < 0.001")

UTD

## Simple Linear Regression for presence of Mul by GA

Mul_Reg <- read.csv("GA_MUL_Simple_Reg.csv")

LinReg7 <- lm(CAKUT ~ GA, data = Mul_Reg)

Mul <- ggplot(LinReg7, aes(x = GA, y = CAKUT)) +
  geom_point() +
  stat_smooth(method = lm) +
  labs(x = "Gestational Age (weeks)", y = "Mul Prevalence per 1,000") +
  scale_x_continuous(breaks = seq(23, 33, by = 1)) +
  stat_regline_equation(label.y.npc = 0.2, label.x.npc = 0.77, aes(label = ..rr.label..))+
  stat_fit_glance(aes(label = paste("P-value =", signif(..p.value.., digits = 2))), label.x=1,
label.y = 0.1) +
  theme_classic()

Mul

## Simple Linear Regression for presence of NOS by GA

NOS_Reg <- read.csv("GA_NOS_Simple_Reg.csv")

LinReg8 <- lm(CAKUT ~ GA, data = NOS_Reg)

NOS <- ggplot(LinReg8, aes(x = GA, y = CAKUT)) +
```

© 2022 Hays T et al. *JAMA Network Open.*

```
  geom_point() +
  stat_smooth(method = lm) +
  labs(x = "Gestational Age (weeks)", y = "NOS Prevalence per 1,000") +
  scale_x_continuous(breaks = seq(23, 33, by = 1)) +
  stat_regline_equation(label.y.npc = 0.1, label.x.npc = 0.74, aes(label = ..rr.label..))+
  theme_classic() +
  annotate("text", x=31.02, y = 0.1, size = 4, label = "P-value < 0.001")

NOS


ggarrange(Any_CAKUT, AWD, EF, RA, RHD, UTD, Mul, NOS, ncol = 4, nrow = 2)




#### Supplemental Figure 6

## Odds of death or severe illness by category of CAKUT, stratified for isolated,
syndromic

library(ggplot2)

boxLabels = c("Any CAKUT", "AWD", "EF", "RA", "RHD", "UTD", "NOS", "Mul")

# Entering summarized data of ORs and CIs of isolated CAKUT

df_I <- data.frame(yAxis = length(boxLabels):1,
                 boxOdds = c(3.88, 5.64, 1.56, 2.30, 3.30, 4.20, 2.88, 5.51),
                 boxCILow = c(3.67, 2.19, 0.98, 1.59, 2.63, 3.93, 2.49, 3.78),
                 boxCIHigh = c(4.11, 14.56, 2.47, 3.16, 4.13, 4.50, 3.34, 8.02))

df_I$group <- "Isolated CAKUT"

# Entering summarized data of ORs and CIs of syndromic CAKUT
df_S <- data.frame(yAxis = length(boxLabels):1,
                 boxOdds = c(7.26, 7.90, 4.67, 5.99, 12.72, 6.82, 5.58, 19.16),
                 boxCILow = c(6.29, 2.74, 2.05, 3.34, 6.87, 5.71, 3.87, 8.01),
                 boxCIHigh = c(8.37, 22.74, 10.65, 10.74, 23.53, 8.16, 8.05, 45.82))

df_S$group <- "CAKUT with Extrarenal Anomaly"

df_Combo = rbind(df_I,df_S)

# Plot
```

© 2022 Hays T et al. *JAMA Network Open.*

```
p <- ggplot(df_Combo, aes(x = boxOdds, y = yAxis, color=group))

p + geom_vline(aes(xintercept = 1), size = .25, linetype = "dashed") +
  geom_errorbarh(aes(xmax = boxCIHigh, xmin = boxCILow), size = .5, height = .2,
position=ggstance::position_dodgev(height=-0.5)) +
  geom_point(shape = 15, size = 3.5, position=ggstance::position_dodgev(height=-0.5))
+
  theme_classic() +
  labs(color = "") +
  theme(legend.position = c(0.8, 0.3)) +
  theme(panel.grid.minor = element_blank()) +
  scale_y_continuous(breaks = seq(1,8,1), labels = c("Mul", "NOS", "UTD", "RHD", "RA",
"EF", "AWD", "Any CAKUT")) +
  ylab("") +
  xlab("Odds Ratio (95% Confidence Interval; Log Scale)") +
  theme(text = element_text(size = 15)) +
  coord_trans(x = 'log10') +
  scale_x_continuous(breaks = c(1,2.5,5, 7.5,10,100),
                     minor_breaks = NULL,
                     labels = c(1,2.5,5, 7.5,10,100))
```

```
#### e Figure 6

library(ggplot2)

# Create labels
boxLabels = c("Death", "ROP", "AKI", "ICH","NEC", "BPD", "Sepsis", "Shock")

# Entering summarized data of ORs and CIs of isolated CAKUT
df1 <- data.frame(yAxis = length(boxLabels):1,
                  boxOdds = c(1.80, 5.00, 1.83, 2.17, 4.44, 3.99, 4.42, 2.29),
                  boxCILow = c(1.63, 4.58, 1.63, 1.94, 3.89, 3.47, 4.15, 2.12),
                  boxCIHigh = c(1.99, 5.46, 2.05, 2.42, 5.06, 4.59, 4.71, 2.46))

df1$group <- "Isolated CAKUT"

# Entering summarized data of ORs and CIs of islated CHD
df2 <- data.frame(yAxis = length(boxLabels):1,
```

© 2022 Hays T et al. *JAMA Network Open.*

```
                boxOdds = c(2.11, 2.02, 1.58, 1.81, 2.52, 1.29, 1.68, 1.90),
                boxCILow = c(1.92, 1.78, 1.39, 1.60, 2.12, 1.02, 1.54, 1.76),
                boxCIHigh = c(2.32, 2.30, 1.79, 2.05, 2.98, 1.63, 1.83, 2.06))

df2$group <- "Isolated Cardiac Anomaly"

# Entering summarized data of ORs and CIs of islated CNS anoms
df3 <- data.frame(yAxis = length(boxLabels):1,
                boxOdds = c(2.97, 3.32, 6.64, 1.94, 5.52, 3.56, 2.29, 2.63),
                boxCILow = c(2.68, 2.92, 6.07, 1.68, 4.76, 2.96, 2.09, 2.41),
                boxCIHigh = c(3.29, 3.78, 7.27, 2.25, 6.41, 4.28, 2.51, 2.87))

df3$group <- "Isolated Neurologic Anomaly"

# Entering summarized data of ORs and CIs of Syndromic cases
df5 <- data.frame(yAxis = length(boxLabels):1,
                boxOdds = c(8.20, 7.24, 3.45, 2.84, 10.84, 3.48, 2.91, 5.05),
                boxCILow = c(7.42, 6.41, 3.00, 2.43, 9.39, 2.72, 2.61, 4.59),
                boxCIHigh = c(9.05, 8.17, 3.97, 3.33, 12.52, 4.34, 3.25, 5.56))

df5$group <- "Multiple Anomalies"




df6 = rbind(df1,df2,df3,df5)

# Plot

p <- ggplot(df6, aes(x = boxOdds, y = yAxis, color=group))

p + geom_vline(aes(xintercept = 1), size = .25, linetype = "dashed")+
  geom_errorbarh(aes(xmax = boxCIHigh, xmin = boxCILow), size = .5, height = .2,
position=ggstance::position_dodgev(height=-.1)) +
  geom_point(shape = 15, size = 3.5, position=ggstance::position_dodgev(height=-.1)) +
  theme_classic() +
  scale_fill_brewer(palette="Blues") +
  labs(color = "")+
  theme(legend.position = c(0.8, 0.65)) +
  theme(panel.grid.minor = element_blank()) +
  scale_y_continuous(breaks = seq(1,8,1), labels = c("Shock", "Sepsis", "ROP", "BPD",
"NEC", "ICH", "AKI", "Death")) +
  ylab("") +
  xlab("Odds Ratio (95% Confidence Interval)")+
  theme(text = element_text(size = 15)) +
  scale_x_continuous(breaks = c(1,2.5,5, 7.5,10),
```

© 2022 Hays T et al. *JAMA Network Open.*

```
minor_breaks = NULL,
labels = c(1,2.5,5, 7.5,10))
```

© 2022 Hays T et al. *JAMA Network Open.*

eTable 1. Prevalence of CAKUT by gestational age and subdivided by CAKUT category.

	No. Affected by CAKUT (prevalence per 1,000)						
Gestational Age (weeks)	AWD	EF	RA	RHD	UTD	Mul	NOS
23	1 (0.12)	7 (0.84)	0 (0)	6 (0.72)	177 (21.35)	4 (0.48)	38 (4.58)
24	0 (0)	6 (0.39)	5 (0.33)	9 (0.59)	341 (22.7)	3 (0.19)	68 (4.52)
25	2 (0.11)	6 (0.34)	4 (0.23)	10 (0.58)	431 (25.11)	7 (0.4)	79 (4.6)
26	1 (0.05)	9 (0.46)	6 (0.31)	20 (1.03)	450 (23.33)	9 (0.46)	97 (5.03)
27	1 (0.04)	12 (0.53)	15 (0.66)	24 (1.06)	472 (20.88)	16 (0.7)	83 (3.67)
28	4 (0.14)	12 (0.43)	17 (0.61)	36 (1.31)	467 (17.02)	16 (0.58)	101 (3.68)
29	6 (0.19)	9 (0.29)	19 (0.62)	49 (1.6)	479 (15.7)	22 (0.72)	90 (2.95)
30	5 (0.12)	15 (0.38)	22 (0.55)	50 (1.27)	506 (12.86)	22 (0.55)	113 (2.87)
31	10 (0.19)	24 (0.47)	35 (0.69)	60 (1.19)	572 (11.35)	24 (0.47)	125 (2.48)
32	15 (0.19)	28 (0.36)	70 (0.91)	102 (1.33)	818 (10.72)	50 (0.65)	175 (2.29)
33	11 (0.1)	27 (0.26)	73 (0.7)	143 (1.38)	956 (9.23)	66 (0.63)	230 (2.22)

Abbreviations: AWD, abdominal wall defects; EF, ectopy or fusion; RA, unilateral or bilateral agenesis; RHD, renal hypoplasia or dysplasia; UTD, urinary tract dilation and anomalies of the urethra or ureters; Mul, multiple different forms of CAKUT; NOS, forms of CAKUT not otherwise specified.

© 2022 Hays T et al. *JAMA Network Open.*

eTable 2. Summary of multiple logistic regression for death or severe illness given individual characteristics.

Characteristic	OR (95% CI)	P-value
Discharge Year		
2000–2004	—	
2005–2009	0.94 (0.91, 0.97)	<0.001
2010–2014	0.68 (0.66, 0.70)	<0.001
2015–2020	0.58 (0.56, 0.60)	<0.001
Antenatal Steroids	0.73 (0.71, 0.75)	<0.001
Sex		
Female	—	
Ambiguous	5.87 (4.02, 8.58)	<0.001
Male	1.32 (1.29, 1.35)	<0.001
Race		
White	—	
Asian	0.97 (0.91, 1.04)	0.428
Black	0.89 (0.87, 0.91)	<0.001
Hispanic	1.22 (1.18, 1.25)	<0.001
Other	0.94 (0.90, 0.99)	0.009
Gestational Age (weeks)	0.64 (0.63, 0.64)	<0.001
Birthweight (Z-score)	0.78 (0.77, 0.79)	<0.001
MV within 72 hours	3.13 (3.06, 3.21)	<0.001
Genetic Disorder	3.30 (2.97, 3.67)	<0.001
Extrarenal Anomaly	3.89 (3.70, 4.10)	<0.001
CAKUT	3.96 (3.70, 4.24)	<0.001

eTable 3. Summary of multiple logistic regression for death or severe illness given individual characteristics as mixed effect model with cases nested in NICU facilities.

Characteristic	OR (95% CI)	P-value
Discharge Year		
2000–2004	—	
2005–2009	0.99 (0.99, 1.00)	<0.001
2010–2014	0.96 (0.95, 0.96)	<0.001
2015–2020	0.94 (0.94, 0.95)	<0.001
Antenatal Steroids	0.96 (0.96, 0.96)	<0.001
Sex		
Female	—	
Ambiguous	1.29 (1.23, 1.35)	<0.001
Male	1.03 (1.03, 1.03)	<0.001
Race		
White	—	
Asian	1.00 (0.99, 1.00)	0.380
Black	0.99 (0.98, 0.99)	<0.001
Hispanic	1.00 (1.00, 1.00)	0.537
Other	0.99 (0.99, 1.00)	0.004
Gestational Age (weeks)	0.93 (0.93, 0.93)	<0.001

© 2022 Hays T et al. *JAMA Network Open.*

	No. Affected (Prevalence per 1000)		
**Genetic Disorder**	**CAKUT Absent**	**CAKUT Present**	**OR (95% CI)**
**Any Genetic Disorder**	3038 (7.56)	348 (43.00)	5.89 (5.26, 6.60)
**Any Aneuploidy**	2514 (6.26)	303 (37.44)	6.17 (5.47, 6.97)
Trisomy 13	114 (0.28)	36 (4.45)	15.74 (10.81, 22.90)
Trisomy 18	204 (0.51)	25 (3.09)	6.10 (4.02, 9.24)
Trisomy 21	1361 (3.39)	111 (13.72)	4.09 (3.37, 4.97)
Trisomy 22	3 (0.01)	0	0
Klinefelter Syndrome	42 (0.10)	3 (0.37)	3.55 (1.10, 11.44)
Turner Syndrome	52 (0.13)	7 (0.86)	6.69 (3.04, 14.72)
Unspecified Aneuploidy	738 (1.84)	121 (14.95)	8.24 (6.79, 10.00)
**Any CNV**	135 (0.34)	21 (2.59)	7.74 (4.88, 12.26)
13q Deletion Syndrome	11 (0.03)	0	0
Cri-du-chat Syndrome	19 (0.05)	3 (0.37)	7.84 (2.32, 26.49)
22q11 Deletion Syndrome	60 (0.15)	9 (1.11)	7.45 (3.70, 15.02)
Unspecified CNV	45 (0.11)	9 (1.11)	9.93 (4.86, 20.33)
**Any Single Gene Disorder**	389 (0.97)	24 (2.97)	3.07 (2.03, 4.64)
Cystic Fibrosis	261 (0.65)	17 (2.10)	3.24 (1.98, 5.29)
Fragile X Syndrome	6 (0.01)	2 (0.25)	16.55 (3.34, 81.99)
Hypophosphatasia	122 (0.30)	5 (0.62)	2.03 (0.83, 4.98)

Birthweight (Z-score)	0.97 (0.97, 0.97)	<0.001
MV within 72 hours	1.17 (1.16, 1.17)	<0.001
Genetic Disorder	1.19 (1.17, 1.20)	<0.001
Extrarenal Anomaly	1.21 (1.20, 1.22)	<0.001
CAKUT	1.21 (1.20, 1.22)	<0.001

eTable 4. Genetic disorders in infants with and without CAKUT.

© 2022 Hays T et al. *JAMA Network Open.*

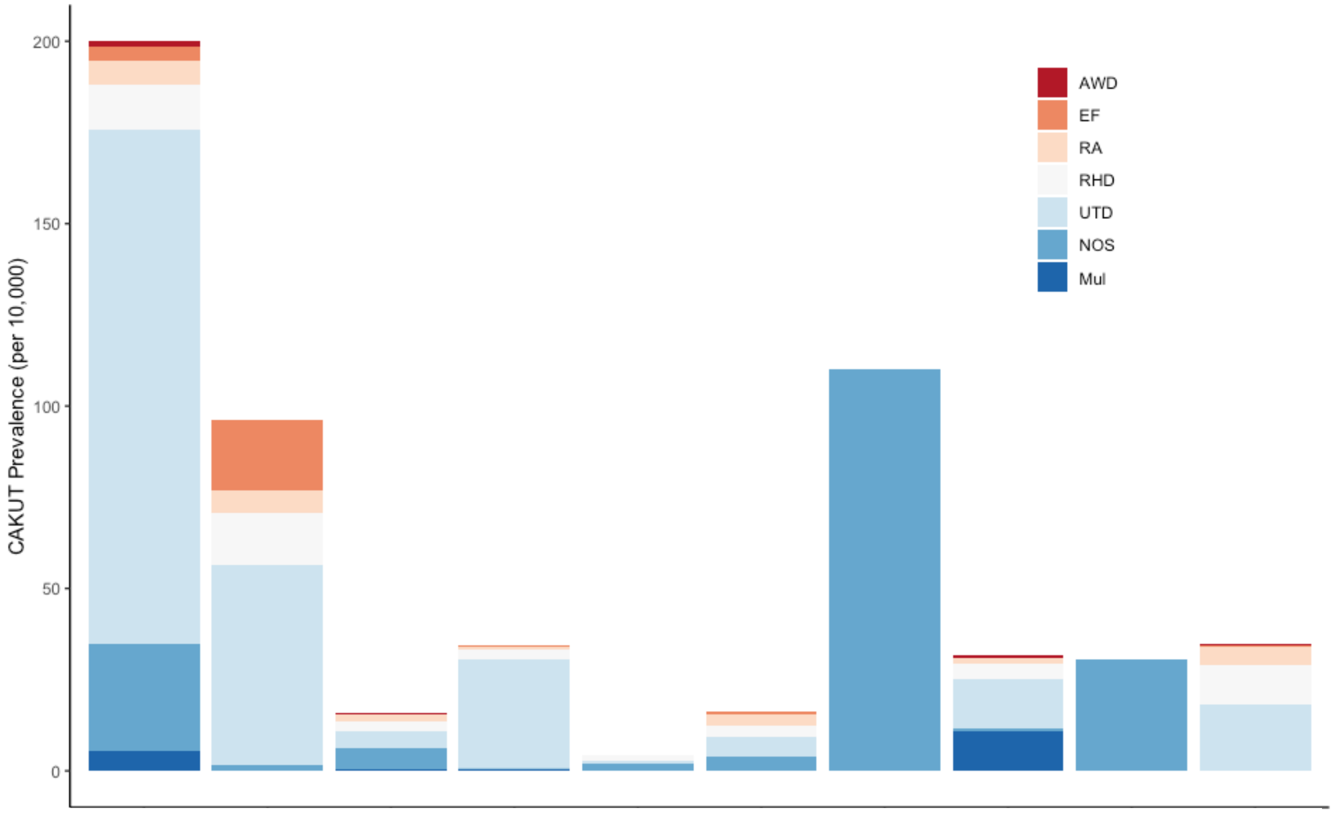

Cohort	Present Study	Ref. 9	Ref. 10	Ref. 11	Ref. 12	Ref. 13	Ref. 14	Ref. 15	Ref. 16	Ref. 17
Descritpion	Preterm Infants	Ultrasound screen of healthy infants	Routine 2nd trimester ultrasound data	Routine 3rd trimester ultrasound data	Registry of live births	Routine 3rd trimester ultrasound data	Routine 2nd and 3rd trimester ultrasound data	Routine 2nd and 3rd trimester ultrasound data	Registry of live births, stillbirths, and terminations	Routine 2nd trimester ultrasound data
Cohort Size	409,704	17,783	709,030	50,193	1,603,794	1,748,038	27,700	4,200,000	223,777	43,209
CAKUT Prevalence (per 10,000)	197.5	96.2	16.0	34.3	4.2	16.0	110.1	31.6	30.4	34.9
OR(95%CI) of CAKUT compared to reference populations	-	2.1 (1.8,2.4)	12.6 (11.9,13.5)	5.9 (5.0,6.8)	48.5 (44.8,52.4)	12.6 (12.1,13.2)	1.8 (1.6,2.0)	6.4 (6.2,6.6)	6.6 (6.1,7.2)	5.8 (4.9,6.8)

eFigure 1. Prevalence of CAKUT in this cohort and in published studies of the general population. Prior studies of the prevalence of CAKUT have found rates ranging from 4 to 110 per 10,000 individuals. These rates, as well as that found in the present study are provided above after stratification by CAKUT category. Given differences in methods of detection, diagnostic criteria, and CAKUT definitions, direct comparisons are limited. The table below describes characteristics of these studies as well as comparative odds ratio of CAKUT prevalence of this cohort of preterm infants compared to these studies of the general population. AWD, abdominal wall defects; EF, ectopy or fusion; Mul, multiple different forms of CAKUT; RA, unilateral or bilateral renal agenesis; RHD, renal hypoplasia or dysplasia; NOS, forms of CAKUT not otherwise specified; UTD urinary tract dilation and anomalies of the urethra or ureters.

© 2022 Hays T et al. *JAMA Network Open.*

Variable		No.	OR (95% CI)
**Sex**			
	**Female**	191105	Reference
	**Ambiguous or Unknown**	305	6.87 (4.78, 9.64)
	**Male**	218294	1.83 (1.75, 1.92)
**Race**			
	**White**	194232	Reference
	**Asian**	11565	1.15 (1.01, 1.30)
	**Black**	93440	0.69 (0.65, 0.73)
	**Hispanic**	79580	1.20 (1.13, 1.26)
	**Other**	30887	1.05 (0.97, 1.15)
**Gestational Age (weeks)**		409704	0.91 (0.91, 0.92)
**Birthweight (Z score)**		409704	0.97 (0.95, 0.99)
**Genetic Disorder**			
	**Absent**	406307	Reference
	**Present**	3397	3.03 (2.67, 3.42)
**Extrarenal Anomaly**			
	**Absent**	388345	Reference
	**Cardiac**	7690	2.70 (2.42, 3.00)
	**Gastrointestinal**	5059	3.94 (3.53, 4.40)
	**Hydrops**	922	4.11 (3.15, 5.28)
	**Multiple Systems**	2034	10.93 (9.67, 12.33)
	**Neurologic**	5076	3.67 (3.27, 4.11)
	**Pulmonary**	578	3.51 (2.46, 4.84)

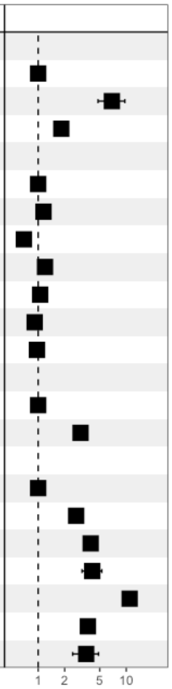

eFigure 2. Multiple logistic regression for presence of CAKUT given individual characteristics. CAKUT was significantly associated with male or ambiguous sex, lower gestational age, lower birthweight (as a Z score stratified by gestational age within this cohort), and with known genetic disorders and extrarenal anomalies. CAKUT also differed by race.

© 2022 Hays T et al. *JAMA Network Open.*

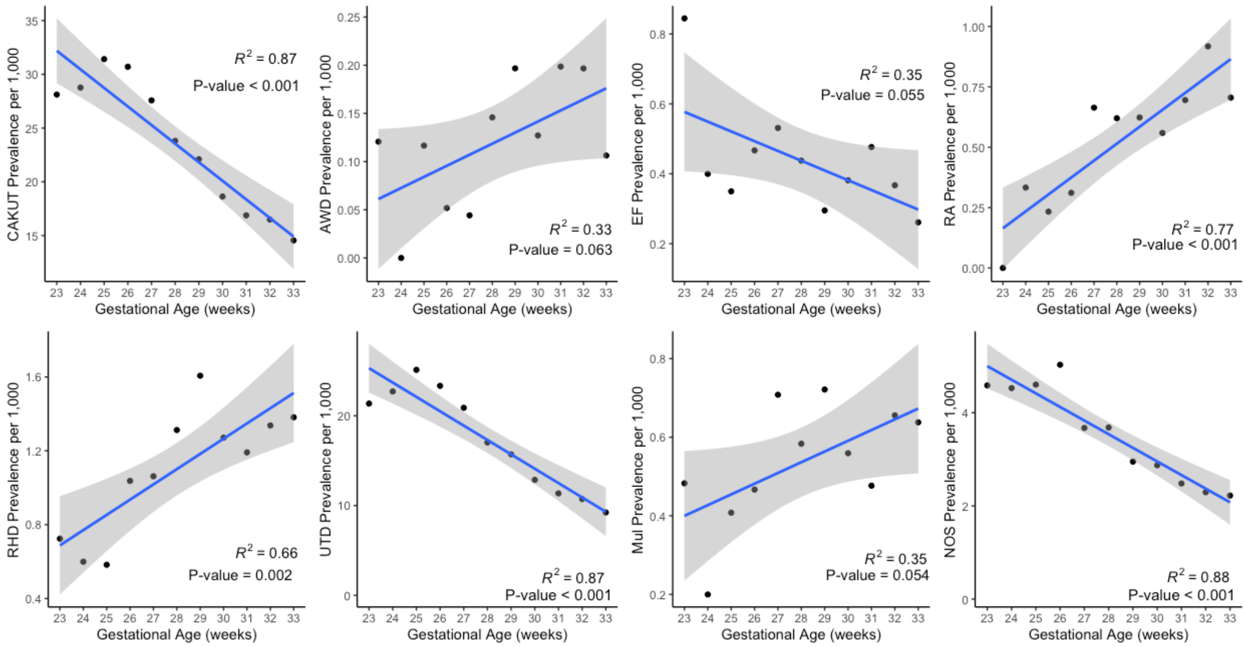

eFigure 3. Correlation between gestational age and prevalence of CAKUT for each category of anomaly. The prevalence of CAKUT is plotted as a function of gestational age in weeks with a simple regression and 95% confidence interval. Significantly correlated positive relationships were found for RA, RHD, and Mul anomalies. Significantly correlated negative relationships were found for UTD and NOS anomalies, as well as for the presence of any form of CAKUT.

© 2022 Hays T et al. *JAMA Network Open.*

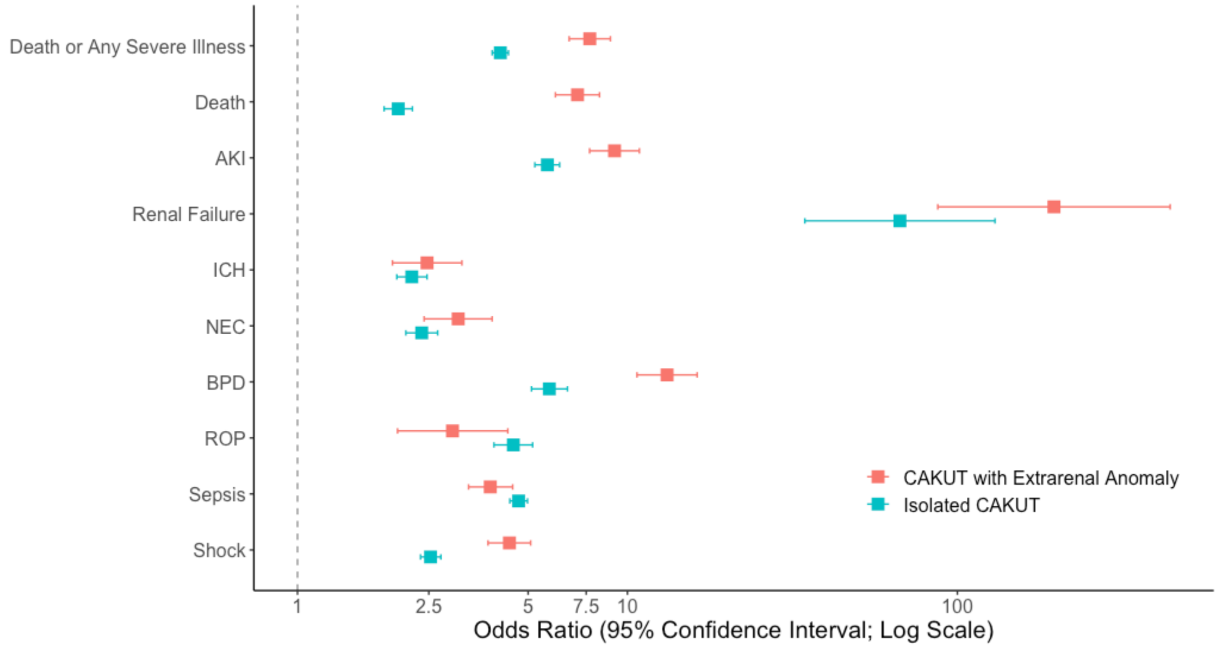

eFigure 4. Odds of severe illness in preterm infants with CAKUT. Death or severe illnesses were stratified by specific diagnoses. The presence of CAKUT, isolated or with extrarenal anomalies, nonspecifically was associated with increased odds of every severe illness analyzed. AKI, acute kidney injury; ICH, intracranial hemorrhage; NEC, necrotizing enterocolitis; BPD, bronchopulmonary dysplasia; ROP, retinopathy of prematurity.

© 2022 Hays T et al. *JAMA Network Open.*

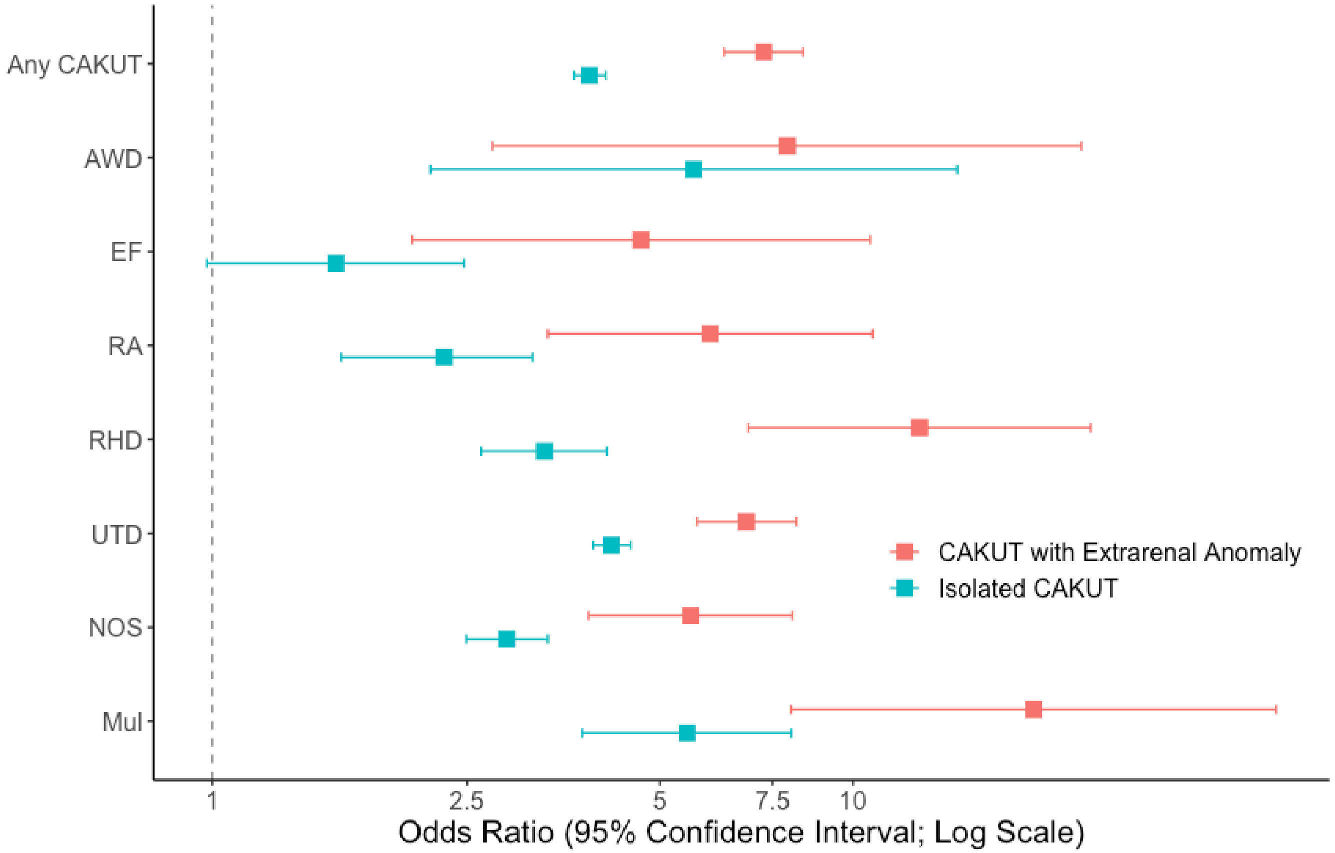

eFigure 5. Odds of death or severe illness in preterm infants with CAKUT stratified by CAKUT categorization. CAKUT, whether in isolation or accompanied by extrarenal anomalies, was associated with increased risk of disease.

© 2022 Hays T et al. *JAMA Network Open.*

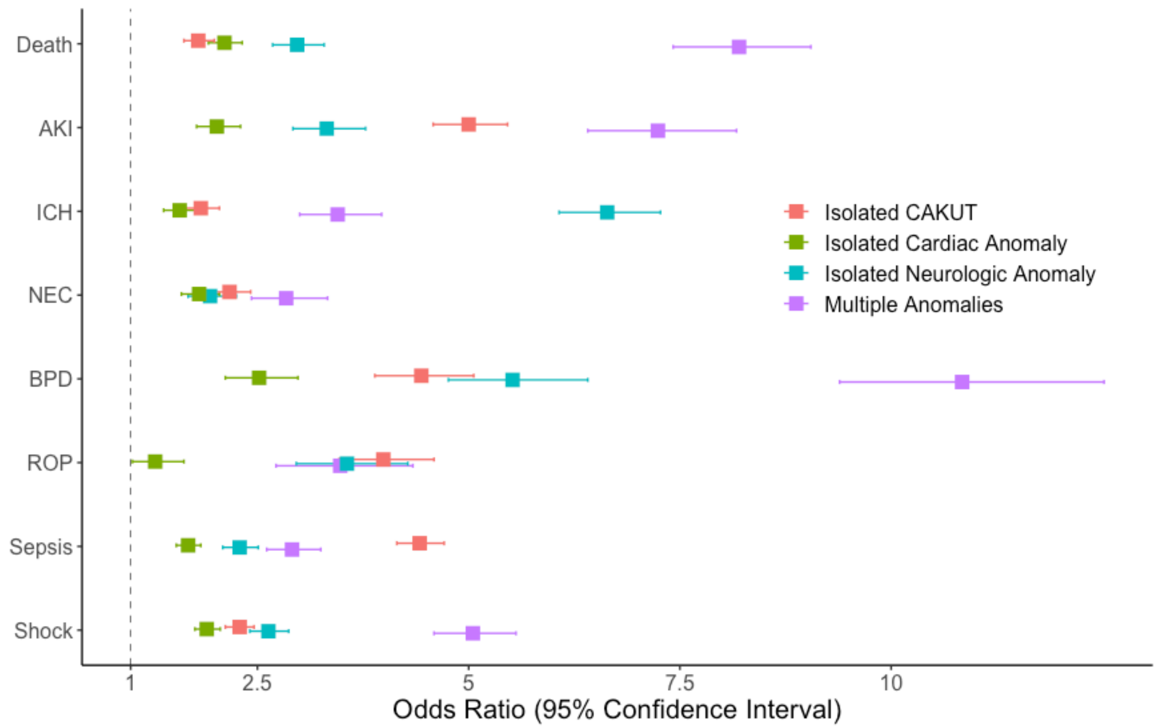

eFigure 6. Odds of death or severe illness in preterm infants with CAKUT and other congenital anomalies. Infants with CAKUT had similar rates of illness as those with isolated cardiac and neurologic anomalies or those with multiple anomalies. AKI, acute kidney injury; ICH, intracranial hemorrhage; NEC, necrotizing enterocolitis; BPD, bronchopulmonary dysplasia; ROP, retinopathy of prematurity.

© 2022 Hays T et al. *JAMA Network Open.*

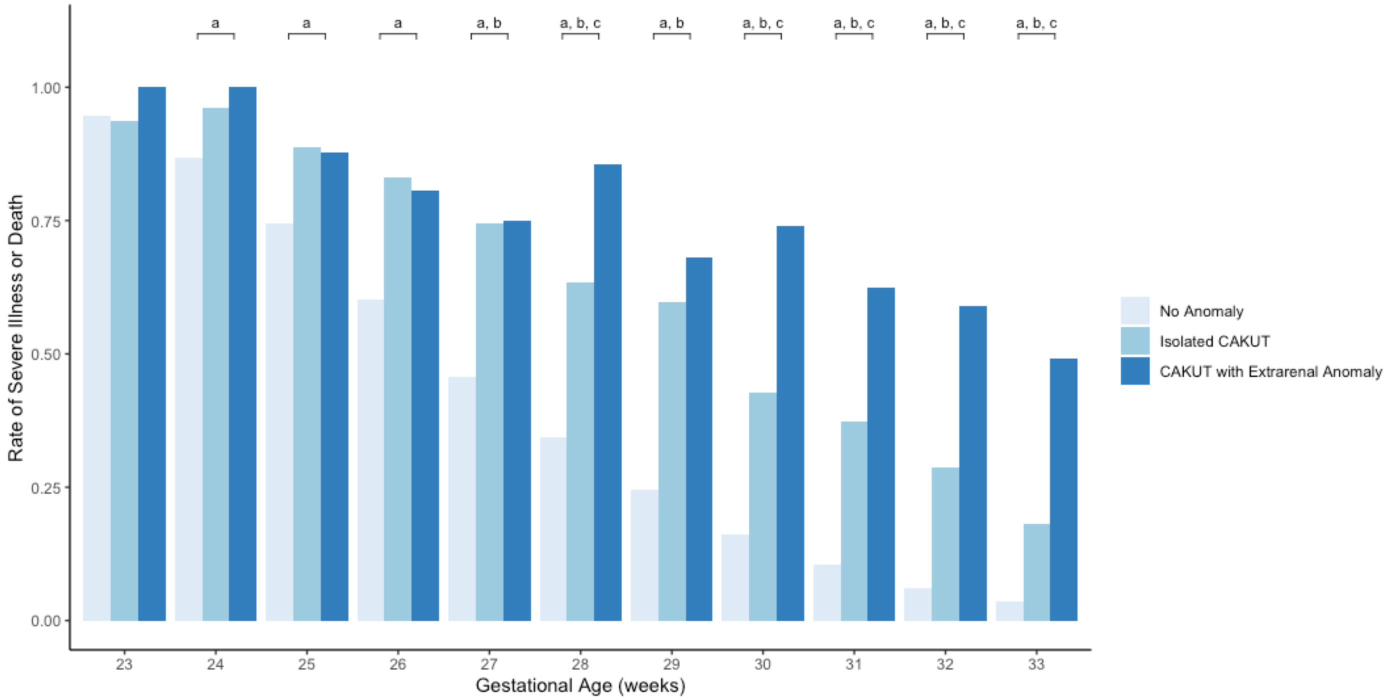

eFigure 7. Rate of death or severe illness by gestational age in infants with and without CAKUT. The presence of CAKUT, in isolation or with extrarenal anomalies, was associated with increased risk of severe illness or death across gestational ages. Statistically significant differences (determined by the Bonferroni-adjusted P-values for pairwise chi squared comparisons at each gestational age) are indicated in brackets. [a]$P < 0.05$, no anomaly vs. isolated CAKUT. [b]$P < 0.05$, no anomaly vs. CAKUT with extrarenal features. [c]$P < 0.05$, isolated CAKUT vs. CAKUT with extrarenal features.

© 2022 Hays T et al. *JAMA Network Open.*
